# The Impact of Pharmacist Vaccination Privilege during a Nation-Wide Measles Outbreak

**DOI:** 10.3390/pharmacy8010007

**Published:** 2020-01-09

**Authors:** Tanya Singh, Renae L. Smith-Ray, Michael Taitel

**Affiliations:** Walgreen Co., Deerfield, IL 60012, USA; renae.smith-ray@walgreens.com (R.L.S.-R.); michael.taitel@walgreens.com (M.T.)

**Keywords:** measles, MMR vaccine, pharmacist vaccination privilege, policy, outbreak, pharmacy, vaccine hesitancy

## Abstract

The 2019 measles outbreak was the worst since the USA eliminated measles in 2000. This paper presents the vaccination trends for a large chain-pharmacy, Walgreens, and examines the estimated vaccination capacity and impact of pharmacist privilege policies across states. Specifically, we estimated the number of people who could have been vaccinated in eight states with reduced or no measles, mumps, and rubella (MMR) vaccination privilege during the study period January–June, 2019. During the study period, Walgreens pharmacists administered MMR vaccines to 62,526 patients, a 231.9% increase. If pharmacists had been permitted to vaccinate against measles in the eight states investigated, Walgreens pharmacies would have administered between 12,404 and 36,551 additional vaccinations during that time. We also estimated all chain pharmacies’ capacity to vaccinate in one state that was severely impacted by the measles outbreak, New York, using a range from normal pharmacy operating conditions to maximum capacity. Assuming sufficient demand, it was estimated that chain pharmacies in New York State would have the capacity to vaccinate between 47,688 and 174,856 patients daily, achieving MMR vaccination (first dose) of the measles-susceptible population within 8–28 days. Overall, this study demonstrates the public health value of pharmacist vaccination privilege during a nation-wide outbreak of measles.

## 1. Introduction

Measles is one of the most contagious infectious diseases, causing illness in 90% of susceptible individuals exposed to an infected patient [1]. For many patients, measles is limited to moderate symptoms of fever, cough, stuffy nose, conjunctivitis, and a maculopapular rash; however, severe complications such as pneumonia or encephalitis can occur [1,2]. Complications are more common in young children, and 1–3 out of 1000 who get measles will die [1].

The combination of measles’ high transmissibility and high risk of disease-driven complications and death motivated the development of a measles vaccine. The vaccine was introduced in 1967, and in the 1970s was combined with mumps and rubella to create the measles, mumps, and rubella (MMR) vaccine [2]. One dose of the vaccine is 93% effective in preventing measles [1]. A second dose was later added to the vaccination recommendation to combat primary vaccine failure. With a combined effectiveness of 97%, the vaccine led to measles being declared eliminated, as defined as the sustained interruption of indigenous transmission, in the United States in 2000 [1]. While outbreaks still occur, the vast majority are associated with travelers bringing measles to the USA from other countries [2].

In recent years, vaccine hesitancy has increased the number of Americans, particularly children, who are unvaccinated or under-vaccinated against the measles, resulting in the illness spreading in the USA from imported cases [3]. Imported cases come from unvaccinated USA citizens or residents who are infected with measles while traveling abroad and return to under-vaccinated communities [3]. Prior to 2019, the highest number of measles cases reported in a calendar year since elimination was 667 cases in 2014 [4]. In 2018, 82 travelers brought measles into the USA, many from under-vaccinated subpopulations [4]. This resulted in a nation-wide outbreak of 1243 cases in 2019 (1 January through 26 September), the largest number of measles cases since 1992 [4].

Pharmacies are well positioned to counter such outbreaks of vaccine-preventable diseases due to their accessible locations and convenient off-clinic hours [5]. Since pharmacists’ scope of practice expanded in the 1990s to include administration of vaccinations, the number of patients who choose to get vaccinated at pharmacies has continued to grow [5,6]. During the 2014–2015 flu season, 22.2% of those receiving the seasonal influenza vaccine had the vaccine administered at a pharmacy [7]. Although laws in all fifty USA states now recognize pharmacists as immunizers, there is considerable variability in the state-specific laws for vaccination privileges. Pharmacists can administer the MMR vaccine without a prescription in most states and territories except Missouri, Georgia, North Carolina, Hawaii, and Puerto Rico where a prescription is required to receive vaccination at a pharmacy. In New York, West Virginia, and the District of Columbia, pharmacists are not permitted to administer the vaccine [8].

Most children receive both doses, as is recommended by the Centers for Disease Control and Prevention (CDC), from their pediatrician or general practitioner [2]. American children are required to receive the MMR vaccination for entry into public schools which has contributed to the increase in MMR vaccination rates; however, religious and philosophical exemptions are permitted in most states and private schools may not have the same vaccination requirements [9]. Additionally, anti-vaccine groups have organized in recent years to spread misinformation regarding vaccine safety [9]. Currently, MMR vaccination rates among children is 90.7%, leaving close to one in ten adults and children susceptible to measles. The CDC recommends that all individuals born after 1956, who did not receive the MMR vaccine as a child or did not receive a second dose, and do not have a medical contraindication, should complete the series to gain the maximum protection against measles [1]. Pharmacies play an important role in expanding access to the MMR for measles-susceptible individuals.

This study examined the impact of pharmacist vaccination privilege in providing the MMR vaccination during the 2019 outbreak. In addition, this study sought to estimate the MMR vaccination potential in New York State, where the largest local outbreak occurred but state law did not permit pharmacists to administer the MMR vaccine [4,8].

## 2. Materials and Methods

We conducted a descriptive analysis of the number and location of MMR vaccinations administered at a large nationwide pharmacy chain, Walgreens. The six-month study period spanned from 1 January 2019 to 30 June 2019. The number of vaccinations administered per month and per state were compared to the number of measles cases in the United States reported to the World Health Organization (WHO) as of September, 2019 [10].

In the eight states where pharmacists did not have MMR vaccination privilege or were only allowed to administer the vaccine with a prescription, we estimated the number of individuals who could have been vaccinated during the study period if such limitations were not in place. This estimate was based on passive demand for the vaccine during the study period from states that permit pharmacists to administer the MMR vaccine without a prescription. A low to high range was generated. The conservative estimate was generated by calculating the average number of patients vaccinated per pharmacy chain-wide after excluding pharmacies in the eight states in question [8]. The liberal estimate was calculated by averaging the number of vaccinated individuals per pharmacy in a state with pharmacist MMR vaccination privilege and the second largest number of measles cases, Washington [11].

Next, we sought to approximate pharmacists’ overall capacity to vaccinate the measles-susceptible population. We used New York State to calculate our capacity as they had the largest measles outbreak. To estimate the number of measles susceptible individuals in the state, we used Census and CDC-reported data. The 2018 total state population of New York was multiplied by the Census-reported proportion of people aged 5–64 [12]. The age range reflects frequent age restrictions for vaccinating young children at pharmacies. Additionally, those born before 1957 do not require the vaccination as they are presumed to be immunized through exposure to the measles virus as a child [1,2]. The 5–64 population was then multiplied by the CDC-reported proportion of MMR vaccination non-coverage (<1 dose) among children in New York State (0.932, averaged from 1995 to 2017) [13].

### 2.1. Measles-Susceptible Population in New York State = (Population of the State) * (% Aged 5–64) * (Proportion of MMR Vaccination Non-Coverage)

Using the number of measles-susceptible population in New York State, we estimated pharmacists’ overall capacity to vaccinate these individuals in chain pharmacy locations throughout the state. Walgreens vaccination data was used to assess the hourly vaccination capacity and those rates were then applied to all chain pharmacies. Estimates assumed a sufficient supply of the MMR vaccine and high patient demand. Under normal operations, we assumed a pharmacy could administer a minimum of three MMR vaccines per hour for eight hours daily, seven days per week. We also sought to calculate the maximum capacity for vaccination using the highest documented number of vaccinations distributed in any Walgreens pharmacy chain-wide. During the measles outbreak, a Walgreens pharmacy in Wenatchee, Washington administered 104 vaccines over 6.5 h during a community-based vaccine clinic. A three-hour adjustment was also included to account for extra staff required to operate the clinic and maintain normal operations in the pharmacy. This resulted in an average of 11 vaccines per hour.

### 2.2. Maximum Hourly MMR Vaccination Rate = 104/(6.5 Clinic Hours + 3 Additional Hours of Staffing)

We utilized SAS Enterprise Guide 7.1 (SAS institute, Cary, NC, USA) to retrieve verified pharmacy claims data from Walgreens enterprise data warehouse and conduct descriptive analyses for the model. All pharmacies in the chain were included in the study, spanning over 49 states (excluding North Dakota), the District of Columbia, and Puerto Rico. Vaccinations were counted at the patient-level at the date of the first dose.

## 3. Results

### 3.1. Distribution of Vaccine Administration in Walgreens Pharmacies

From 1 January through 30 June 2019, pharmacists administered MMR vaccines to 62,526 patients. This was a year-over-year (YOY) increase of 231.9% and reflects the increased patient demand in response to the nation-wide measles outbreak (Table 1). The month of May saw the largest YOY increase, 648.3%, with 24,671 patients vaccinated.

**Figure 1:**
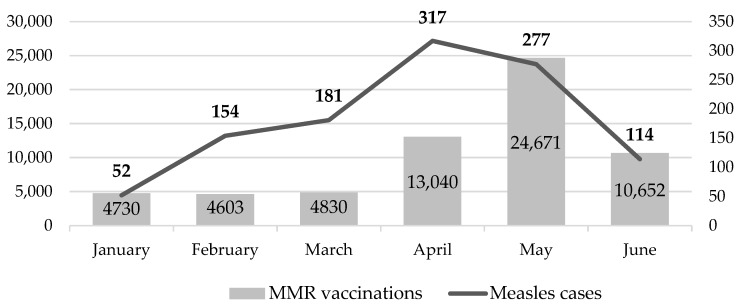
Nation-wide number of reported measles cases and number of patients vaccinated against measles at Walgreens pharmacies in 2019.

When compared to the number of monthly reported measles cases to WHO, the increasing number of vaccinations from February to May lagged one month behind increases in reported cases (Figure 1).

**Figure 2:**
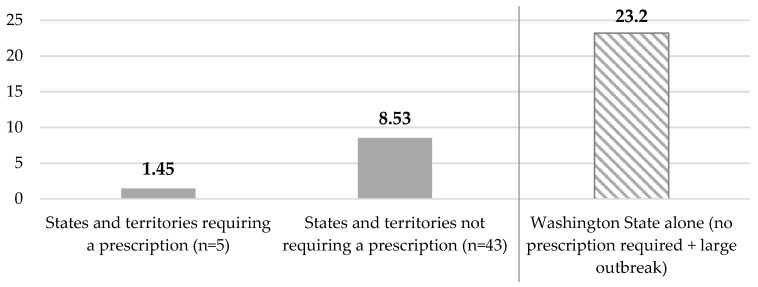
Average number of MMR vaccines administered per Walgreens pharmacy location from 1 January to 30 June 2019.

Over the study period, the average number of MMR vaccines administered per pharmacy in states that require an MMR vaccine prescription (n = 1128) was 1.45; while those which did not require prescription (n = 7139) administered an average of 8.53 per store. Looking at Washington State alone, where the demand for vaccines was much higher due to several significant outbreaks, the average per store (n = 141) was 23.20 (Figure 2).

### 3.2. Estimation of Walgreens Vaccination Opportunity in States with Limited Pharmacist Privilege

Using MMR vaccination averages per Walgreens pharmacy, we estimated that if pharmacists had been allowed to fully administer the MMR vaccine (without requiring a prescription) in the eight states with reduced or no MMR vaccination privilege, Walgreens pharmacies (n = 1646) would have administered between 14,040 and 38,187 MMR doses during the six-month study period, an additional 12,404 to 36,551 vaccinations.

### 3.3. Estimation of the Vaccination Capacity for All Chain Pharmacies in New York State

Approximately 1,298,971 measles-susceptible individuals over the age of five were living in New York State in 2018, based on CDC-reported childhood MMR vaccine non-coverage [13]. The number of chain pharmacy locations in the state totaled 1987 (487 Walgreens and 1500 other chain locations). Under normal operations, we estimated that these pharmacies would have the capacity to administer the vaccine to 47,688 patients daily (24 per pharmacy), assuming sufficient patient demand for MMR vaccination. At maximum capacity, that number would increase up to 174,856 patients daily (88 per pharmacy). Under this scenario, administration of one dose of the MMR vaccine for the entire measles-susceptible population in New York State could be achieved within 8 to 28 days. To complete the series, the second dose can be given as early as four weeks after the first MMR vaccine. If all individuals returned for their second dose by eight weeks, series completion could be achieved within 64 to 84 days.

## 4. Discussion

Our analysis demonstrates how pharmacist vaccination privilege allowed many pharmacists to effectively meet the needs of their communities during a large nation-wide measles outbreak. Modeling the MMR vaccination capacity at pharmacies during this large measles outbreak and understanding the impact of pharmacist privilege can have an impact on policy and future collaborations with local communities. In April 2019, the number of cases were increasing so rapidly in New York City that a temporary emergency order was implemented, mandating all unvaccinated individuals born after 1956 without a medical exemption receive the measles vaccine [2,14]. Individuals were able to go to their primary care provider or public health department for the vaccine; however, pharmacists could have also assisted in this process. [8]. As the most accessible healthcare providers in the United States, community pharmacists can administer vaccinations during convenient off-clinic hours and accommodate walk-in patients [5].

At minimum, pharmacies can help ease the workload of local public health departments and primary care offices by vaccinating patients during outbreaks. They can also provide vaccination clinics in collaboration with local communities. Pharmacies can also contribute during outbreaks by partnering with local governments and health departments to communicate the importance of vaccination, which may help increase vaccination rates and prevent future outbreaks. While the temporary mandate in New York City was in direct response to the public health emergency of the measles outbreak, achieving sustained reductions in vaccine hesitancy requires implementation of evidence-based interventions that include messaging to address concerns and build confidence in vaccines [9]. These messages could be provided digitally or at the counter during a pharmacist consultation.

One limitation to our study was the distinction between patient demand for the MMR vaccine and capacity to vaccinate. The more than three-fold YOY increase in MMR vaccination rates during the study period demonstrates the impact of the measles outbreak and public health messaging on the public’s perception of risk and desire to get vaccinated. Still, realizing the 24 to 88 patients per day capacity in all New York State chain pharmacies would reflect an extremely high demand for the vaccine. It also assumes an even distribution of measles-susceptible individuals between pharmacies across the state.

Another limitation in this study is that we did not have access to patients’ complete vaccination records, and therefore were unable to distinguish between first and second doses of the vaccine series. While some patients acquired both doses at their Walgreens, a majority received only one. It is likely that some of the patients who only received one vaccine at their pharmacy only needed the second dose, or “booster”, because they had received one dose but missed the second during childhood. Others simply did not return to their pharmacy for their second dose, given that the first dose alone is 93% protective against measles [1].

## 5. Conclusions

During the 2019 measles outbreak, Walgreens experienced a significant increase in patient demand for protection and responded by administering a large number of MMR vaccines throughout the United States. The number of vaccinations delivered per pharmacy varied greatly depending on the pharmacist vaccination privileges allowed in the state. Our model showed the potential that exists for pharmacists to respond to vaccine-preventable outbreaks, given the necessary vaccination privileges. The states that do not permit pharmacists to administer the MMR vaccine missed a huge opportunity for pharmacies to assist in protecting population health during the county’s worst measles outbreak since eliminating the disease. Pharmacies are the most accessible healthcare destination and play an important role supporting in public health. The recent measles outbreak and pharmacy response demonstrated the need for required permissions and collaboration by pharmacies and government at all levels to fully realize their vaccination capacity and support population health.

## Figures and Tables

**Table 1:** Number of patients receiving MMR Vaccines at Walgreens in 2018 and 2019 with percent increase.

Month	2018	2019	Percent Increase
January	3361	4730	40.7%
February	2601	4603	77.0%
March	2971	4830	62.6%
April	3150	13040	314.0%
May	3297	24671	648.3%
June	3459	10652	208.0%
**Total**	**18,839**	**62,526**	**231.9%**
